# Association of the Estimated Glomerular Filtration Rate With vs Without a Coefficient for Race With Time to Eligibility for Kidney Transplant

**DOI:** 10.1001/jamanetworkopen.2020.34004

**Published:** 2021-01-14

**Authors:** Leila R. Zelnick, Nicolae Leca, Bessie Young, Nisha Bansal

**Affiliations:** 1 Kidney Research Institute, Division of Nephrology, University of Washington, Seattle; 2 Division of Nephrology, University of Washington, Seattle; 3 Puget Sound Veterans Affairs Health Care System, Seattle, Washington

## Abstract

**Importance:**

Kidney transplant is associated with improved survival and quality of life among patients with kidney failure; however, significant racial disparities have been noted in transplant access. Common equations that estimate glomerular filtration rate (eGFR) include adjustment for Black race; however, how inclusion of the race coefficient in common eGFR equations corresponds with measured GFR and whether it is associated with delayed eligibility for kidney transplant listing are unknown.

**Objective:**

To compare eGFR with measured GFR and evaluate the association between eGFR calculated with vs without a coefficient for race and time to eligibility for kidney transplant.

**Design, Setting, and Participants:**

This prospective cohort study used data from the Chronic Renal Insufficiency Cohort, a multicenter cohort study of participants with chronic kidney disease (CKD). Self-identified Black participants from that study were enrolled between April 2003 and September 2008, with follow-up through December 2018. Statistical analyses were completed on November 11, 2020.

**Exposure:**

Estimated GFR, measured annually and estimated using the creatinine-based Chronic Kidney Disease-Epidemiology (CKD-EPI) equation with and without a race coefficient.

**Main Outcomes and Measures:**

Iothalamate GFR (iGFR) measured in a subset of participants (n = 311) and time to achievement of an eGFR less than 20 mL/min/1.73 m^2^, an established threshold for kidney transplant referral and listing.

**Results:**

Among 1658 self-identified Black participants, mean (SD) age was 58 (11) years, 848 (51%) were female, and mean (SD) eGFR was 44 (15) mL/min/1.73 m^2^. The CKD-EPI eGFR with the race coefficient overestimated iGFR by a mean of 3.1 mL/min/1.73 m^2^ (95% CI, 2.2-3.9 mL/min/1.73 m^2^; *P* < .001). The mean difference between CKD-EPI eGFR without the race coefficient and iGFR was of smaller magnitude (−1.7 mL/min/1.73 m^2^; 95% CI, −2.5 to −0.9 mL/min/1.73 m^2^). For participants with an iGFR of 20 to 25 mL/min/1.73 m^2^, the mean difference in eGFR with vs without the race coefficient and iGFR was 5.1 mL/min/1.73 m^2^ (95% CI, 3.3-6.9 mL/min/1.73 m^2^) vs 1.3 mL/min/1.73 m^2^ (95% CI, −0.3 to 2.9 mL/min/1.73 m^2^). Over a median follow-up time of 4 years (interquartile range, 1-10 years), use of eGFR calculated without vs with the race coefficient was associated with a 35% (95% CI, 29%-41%) higher risk of achieving an eGFR less than 20 mL/min/1.73 m^2^ and a shorter median time to this end point of 1.9 years.

**Conclusions and Relevance:**

In this cohort study, inclusion of the race coefficient in the estimation of GFR was associated with greater bias in GFR estimation and with delayed achievement of a clinical threshold for kidney transplant referral and eligibility. These findings suggest that nephrologists and transplant programs should be cautious when using current estimating equations to determine kidney transplant eligibility.

## Key Points

**Question:**

Is adjusting for Black race in estimating equations for glomerular filtration rate in patients with chronic kidney disease associated with a delay in kidney transplant eligibility?

**Findings:**

In this cohort study of 1658 self-identified Black adults with chronic kidney disease, commonly used estimates of kidney function did not correspond well with directly measured kidney function. Estimating kidney function not including a coefficient for race (vs including a race coefficient) was significantly associated with a shorter time to achieving an estimated glomerular filtration rate less than 20 mL/min/1.73 m^2^, a key threshold of kidney function for referral and listing for kidney transplant.

**Meaning:**

The findings suggest that biases in race-based glomerular filtration rate estimates may be associated with delays in potential kidney transplant eligibility.

## Teaser

This cohort study assesses the association between estimated glomerular filtration rate (eGFR) calculated with vs without a coefficient for race and time to achievement of an eGFR less than 20 mL/min/1.73 m^2^.

## Introduction

Racial disparities in kidney transplantation are well recognized and persistent despite changes in organ allocation in the US.^1^ Black patients are less likely to be referred for transplant, less likely to be wait-listed, and less likely to receive a kidney transplant^2,3^ despite transplant being associated with improved survival and quality of life compared with dialysis.^4^

Patients are considered eligible for kidney transplant evaluation after their glomerular filtration rate (GFR) decreases to less than 20 mL/min/1.73 m^2^. In most cases, the GFR is estimated from equations to determine this transplant eligibility. Recently, the estimating equations for GFR have been debated since the most commonly used estimation applies a race coefficient that increases the estimated GFR (eGFR) for patients identified as Black.^5^ This is controversial for numerous reasons. Race is a complex social construct and is distinct from biological variables, such as ancestry.^6^ Moreover, the coefficient was applied to statistically adjust for non-GFR factors associated with serum creatinine level, including differences in muscle mass in Black persons,^7^ which may not apply to all Black persons, particularly in the context of chronic kidney disease (CKD) and frailty.^8^ Furthermore, definitions of race may be subject to significant bias because race may be self-reported or assigned by a clinician or observer. It is possible that by increasing the estimate of GFR, inclusion of the race coefficient in these equations may contribute to health disparities, including eligibility for kidney transplant, which uses a strict GFR cutoff.

Thus, we hypothesized that eGFR calculated without the race coefficient would correspond more closely with iGFR compared with eGFR calculated with the race coefficient. Furthermore, we hypothesized that estimation of GFR with the race coefficient may be associated with a delay in reaching an eGFR less than 20 mL/min/1.73 m^2^, the threshold for kidney transplant referral, compared with estimation of eGFR without the race coefficient in the same participants with CKD.

## Methods

### Study Population

This cohort study used National Institute of Diabetes and Digestive and Kidney Diseases (NIDDK) public repository data for the Chronic Renal Insufficiency Cohort (CRIC) study,^9^ a multicenter study of 3939 participants with CKD that has been previously described.^10,11^ In brief, the CRIC study enrolled participants at 7 clinical centers from across the US between April 2003 and September 2008, with available follow-up through December 2018. All study participants provided written informed consent, and the study protocol was approved by institutional review boards at each of the participating sites. For the present analysis, we included the 1658 participants who self-identified as Black (eFigure 1 in the Supplement). The present study was determined to be exempt from review by the University of Washington institutional review board because all data were deidentified. This study followed the Strengthening the Reporting of Observational Studies in Epidemiology (STROBE) reporting guideline.

### Estimation of GFR

Serum creatinine level was measured annually at the CRIC study visit using an enzymatic method and isotope dilution mass spectrometry calibrated to a common standard.^12^ Serum cystatin C level was measured using a particle-enhanced immunonephelometric assay; an internal standardization was used to correct for drift over time.^13^ The GFR was estimated annually using several estimating equations: (1) the creatinine-based CKD-EPI equation including the race coefficient (CKD-EPI_RC_)^14^; (2) the same creatinine-based CKI-EPI equation but not including the race coefficient (CKD-EPI_WRC_)^14^; and (3) the cystatin C–based CKD-EPI equation, which does not include a coefficient for race (CKD-EPI_CYS_).^15^

### Measurement of Iothalamate GFR

A total of 1288 CRIC participants were randomly selected to undergo direct measurements of GFR by iothalamate clearance at the baseline study visit.^16^ Additional exclusion criteria for the procedure were known iodine allergy, pregnancy, and impaired urinary voiding. In brief, ^125^I-iothalamate was injected subcutaneously into participants after a water load and administration of a saturated solution of potassium iodine. After a 60- to 90-minute waiting period, timed collections of urine and serum samples were performed, with urine flow rate maintained at greater than 1 mL/min. The goal was to obtain 4 timed urine sample collection periods bracketed by blood sample obtainment to measure plasma iothalamate levels (P); concurrent urine counts (U) and urine volumes (V) were obtained. The GFR was then calculated as the time-weighted mean (U × V)/P and corrected for body surface area; further details have been previously published.^17^

Among the analytic population of self-identified Black CRIC participants, a subset of 311 participants had an iothalamate GFR (iGFR) of 15 to less than 45 mL/min/1.73 m^2^ measured within 60 days of the CRIC study visit at which a blood sample was obtained for calculation of eGFR (eFigure 1 and eTable 1 in the Supplement). Some participants repeated the procedure described above at subsequent study visits, with most repeated measurements occurring at the year-2 and year-4 visits. Among participants with an iGFR measurement of 15 to less than 45 mL/min/1.73 m^2^ (470 total measurements in this range), 185 had 1 measurement, 93 had 2, and 33 had 3; of these, 348 iGFR measurements were obtained the same day as the CRIC study visit.

### Survival Outcomes

The primary survival outcome was time to an eGFR less than 20 mL/min/1.73 m^2^ among participants with a baseline eGFR (CKD-EPI_RC_) of at least 20 mL/min/1.73 m^2^. We calculated the participants’ eGFRs at each visit using the alternative definitions of eGFR (CKD-EPI_RC_, CKD-EPI_WRC_, and CKD-EPI_CYS_). The time until the first eGFR less than 20 mL/min/1.73 m^2^ was calculated as the number of days from study entry to the first study visit at which the eGFR was less than 20 mL/min/1.73 m^2^. We used all available serum creatinine and cystatin C measures from annual study visits during follow-up, censoring for death, end stage kidney disease, loss to follow-up, and end of study. As a sensitivity analysis, we repeated the process among participants with baseline eGFR (CKD-EPI_RC_) of at least 30 mL/min/1.73 m^2^ to calculate the time until the first eGFR less than 30 mL/min/1.73 m^2^, another threshold that may influence medical decision-making in advanced stages of CKD.^18,19,20^

### Covariates

Participants provided information at the baseline study visit about sociodemographic characteristics, medical history, medication use, and lifestyle behaviors. Race/ethnicity was determined by self-report, as was history of cardiovascular disease. Diabetes was defined as a fasting glucose level greater than 126 mg/dL, a nonfasting glucose level greater than 200 mg/dL, or use of insulin or other antidiabetic medications. Smoking status was classified as current, former, or never; blood pressure was obtained by research coordinators in a standardized setting. Body mass index (BMI) was calculated as weight in kilograms divided by height in meters squared from measurements obtained at CRIC study visits.

### Statistical Analysis

Statistical analyses were completed on November 11, 2020. Among the subset of participants with iGFR measurements of 15 to less than 45 mL/min/1.73 m^2^, we compared the bias of estimated eGFR with respect to iGFR using the creatinine-based CKD-EPI equation with and without the race coefficient and the cystatin C–based CKD-EPI equation. As a direct measure of GFR, iGFR was considered to be the gold standard for determining the bias of other estimates of GFR. Because some individuals had multiple iGFR measurements, we used a linear mixed model approach with random intercepts to account for the within-person correlation. We estimated the mean overall and by 5-mL/min/1.73 m^2^ categories of iGFR using intercept-only models; a similar approach was used to estimate the mean difference and associated 95% CIs between measures. As a sensitivity analysis, we examined the bias of eGFR measures across categories of BMI.

We next estimated the association between the estimating equation used to calculate eGFR and the time to achievement of an eGFR less than 20 mL/min/1.73 m^2^. We calculated the times to event that would have occurred for each of the 1616 participants with an eGFR (CKD-EPI_RC_) of at least 20 mL/min/1.73 m^2^ at study entry if the CKD-EPI_RC_, CKD-EPI_WRC_, or CKD-EPI_CYS_ equation had been used to calculate eGFR. We used a discrete Cox proportional hazards regression analysis with infinitesimal jackknife SEs to account for the within-person correlation because each person had 3 times to event.^21^ In a sensitivity analysis, we repeated the Cox proportional hazards regression analysis described above among participants with an eGFR (CKD-EPI_RC_) of at least 30 mL/min/1.73 m^2^ at study entry using the outcome of time to achievement of an eGFR less than 30 mL/min/1.73 m^2^, because this is also an important clinical threshold that may lead to intensification of clinical management. In a second sensitivity analysis, we examined the correspondence of eGFR and iGFR stratified by categories of BMI because a rationale for the race correction for eGFR is to account for differences in body composition. For all analyses, a 2-tailed *P* < .05 was considered to indicate statistical significance. All analyses were conducted using R, version 3.6.2 (R Foundation for Statistical Computing).

## Results

The study population included 1658 self-identified Black participants overall, 848 (51%) of whom were female; the mean (SD) age was 58 (11) years, and the mean (SD) eGFR was 44 (15) mL/min/1.73 m^2^. The median 24-hour urine protein to creatinine ratio was 196 mg/g (interquartile range [IQR], 61-871 mg/g) (Table 1). The majority of participants had an income of less than $50 000 (1069 [64%]) and had at least some college education (850 [51%]); most (1128 [68%]) were insured. Study participants had a mean (SD) follow-up time in the CRIC study of 9.8 (4.1) years.

**Table 1.** Baseline Characteristics of Self-identified Black Participants in the Chronic Renal Insufficiency Cohort Study

Characteristic	Overall (N = 1658)^a^
Follow-up time, y^b^	
Mean (SD)	9.8 (4.1)
Median (IQR)	11.6 (6.6-13.0)
Age, mean (SD), y	57.6 (10.6)
Female	848 (51)
Income, $	
≤20 000	649 (39)
20 001-50 000	420 (25)
50 001-100 000	216 (13)
>100 000	62 (4)
Educational level	
Less than high school	441 (27)
High school graduate	367 (22)
Some college	569 (34)
College graduate or higher	281 (17)
Insurance status	
None	96 (6)
Medicaid or public aid	329 (20)
Any Medicare	496 (30)
VA, military, or CHAMPUS	111 (7)
Private or commercial	192 (12)
Medical history	
Cardiovascular disease	633 (38)
Hypertension	1540 (93)
Myocardial infarction	364 (22)
Congestive heart failure	218 (13)
Atrial fibrillation	328 (20)
Stroke	230 (14)
Diabetes	854 (52)
Blood pressure, mean (SD), mmHg	
Systolic	132.8 (23.1)
Diastolic	73.7 (13.8)
BMI, mean (SD)	33.5 (8.3)
Smoking	321 (19)
eGFR CKD-EPI_RC_, mean (SD), mL/min/1.73m^2^^c^	43.7 (14.9)
24-h Urine protein-to-creatinine ratio, median (IQR), mg/g	196 (61-871)
Serum albumin level, mean (SD), g/dL	3.9 (0.5)
Calcium level, mean (SD), mg/dL	9.2 (0.5)
Phosphate level, mean (SD), mg/dL	3.8 (0.7)

Abbreviations: BMI, body mass index (calculated as weight in kilograms divided by height in meters squared); CHAMPUS, Civilian Health and Medical Program of the Uniformed Services; CKD, chronic kidney disease; CKD-EPI_RC_, creatinine-based CKD-EPI eGFR calculated with the race coefficient; eGFR, estimated glomerular filtration rate; IQR, interquartile range; VA, Veterans Affairs.

SI conversion: To convert serum albumin level to g/L, multiply by 10; calcium level to mmol/L, multiply by 0.25; and phosphate level to mmol/L, multiply by 0.323.

^a^ Data are presented as number (%) of individuals unless otherwise indicated.

^b^ CKD-EPI_RC_ eGFR is the eGFR calculated using the creatinine-based Chronic Kidney Disease Epidemiology Collaboration equation including the race coefficient.

^c^ Follow-up time is the time in years of follow-up in the Chronic Renal Insufficiency Cohort study, censoring for death, loss to follow-up, or end of study.

### Correspondence of iGFR With eGFR Measurements With and Without the Race Coefficient

Among the Black participants in the study, the strongest correlation with iGFR was seen with eGFR CKD-EPI_WRC_ (*r* = 0.75) compared with eGFR CKD-EPI_RC_ (*r* = 0.61) or eGFR_cys_ (*r* = 0.03) (eFigure 2 in the Supplement). In the subset of Black participants with an iGFR of 15 to less than 45 mL/min/1.73 m^2^, the mean (SD) iGFR was 31.8 (6.3) mL/min/1.73 m^2^, the mean (SD) eGFR (CKD-EPI_RC_) was 34.9 (7.8) mL/min/1.73 m^2^, and the mean (SD) eGFR (CKD-EPI_WRC_) was 30.1 (6.7) mL/min/1.73 m^2^ (Table 2). Use of the race coefficient in the creatinine-based CKD-EPI equation resulted in an eGFR that was a mean of 4.8 mL/min/1.73 m^2^ (95% CI, 4.6-4.9 mL/min/1.73 m^2^) higher than that when no race coefficient was used. The CKD-EPI_RC_ estimates of GFR overestimated iGFR by a mean of 3.1 mL/min/1.73m^2^ (95% CI, 2.2-3.9 mL/min/1.73 m^2^; *P* < .001), with similar mean differences across most iGFR categories. In contrast, the mean difference between CKD-EPI_WRC_ and iGFR was of smaller magnitude overall (−1.7 mL/min/1.73 m^2^; 95% CI, −2.5 to −0.9 mL/min/1.73 m^2^); for participants with an iGFR of 20 to less than 35 mL/min/1.73 m^2^, the mean difference (eGFR [CKD-EPI_WRC_] − iGFR) was not statistically significant. For participants with an iGFR of 20 to 25 mL/min/1.73 m^2^, the mean difference in eGFR with vs without the race coefficient and iGFR was 5.1 mL/min/1.73 m^2^ (95% CI, 3.3-6.9 mL/min/1.73 m^2^) vs 1.3 mL/min/1.73 m^2^ (95% CI, −0.3 to 2.9 mL/min/1.73 m^2^). The eGFR (CKD-EPI_CYS_) also significantly overestimated iGFR for participants with an iGFR of 15 to less than 45 mL/min/1.73 m^2^ by a mean of 5.6 mL/min/1.73 m^2^ (95% CI, 4.6-6.6 mL/min/1.73 m^2^). In a sensitivity analysis, results were largely consistent across categories of BMI (eTable 2 in the Supplement).

**Table 2.** Correspondence of eGFR and iGFR Among All Self-identified Black Participants With an iGFR of 15 to Less Than 45 mL/min/1.73 m^2^ in the Chronic Renal Insufficiency Cohort Study

	iGFR, mL/min/1.73 m^2^													
	Overall	*P* value	15 to <20	*P* value	20 to <25	*P* value	25 to <30	*P* value	30 to <35	*P* value	35 to <40	*P* value	40 to <45	*P* value
Measurements, No.	470	NA	37	NA	76	NA	87	NA	84	NA	88	NA	98	NA
Unique individuals, No.	311	NA	20	NA	44	NA	56	NA	58	NA	56	NA	77	NA
Measurement, mean (SD), mL/min/1.73 m^2^^a^														
iGFR	31.8 (6.3)	NA	17.9 (1.5)	NA	22.6 (1.5)	NA	27.7 (1.5)	NA	32.6 (0.9)	NA	37.3 (1.4)	NA	42.6 (1.4)	NA
CKD-EPI_RC_	34.9 (7.8)	NA	22.7 (4.2)	NA	27.9 (4.1)	NA	30.6 (6.8)	NA	36.4 (3.9)	NA	40.8 (7)	NA	42.9 (4.6)	NA
CKD-EPI_WRC_	30.1 (6.7)	NA	19.6 (3.6)	NA	24.1 (3.5)	NA	26.4 (5.8)	NA	31.4 (3.4)	NA	35.2 (6)	NA	37 (4)	NA
CKD-EPI_CYS_	37.4 (8.3)	NA	24.4 (4.1)	NA	28.2 (9.1)	NA	32.2 (6.3)	NA	38.5 (4.3)	NA	45 (8.7)	NA	46.9 (6)	NA
Difference in measurements, mean (95% CI), mL/min/1.73 m^2^^b^														
CKD-EPI_RC_ – CKD-EPI_WRC_	4.8 (4.6 to 4.9)	<.001	3.1 (2.8 to 3.5)	<.001	3.8 (3.6 to 4.1)	<.001	4.2 (4.0 to 4.4)	<.001	5.0 (4.7 to 5.2)	<.001	5.6 (5.4 to 5.9)	<.001	5.8 (5.6 to 6.1)	<.001
CKD-EPI_RC_ – CKD-EPI_CYS_	−2.5 (−3.5 to −1.5)	<.001	−2.2 (−4.7 to 0.3)	.09	−1.4 (−3.3 to 0.4)	.12	−1.5 (−3.1 to 0.2)	.09	−1.6 (−3.3 to 0.1)	.07	−3.6 (−5.3 to −1.9)	<.001	−4.1 (−5.7 to −2.5)	<.001
CKD-EPI_RC_ − iGFR	3.1 (2.2 to 3.9)	<.001	4.8 (2.3 to 7.4)	<.001	5.1 (3.3 to 6.9)	<.001	2.8 (1.2 to 4.5)	<.001	3.7 (2.0 to 5.4)	<.001	3.7 (2.0 to 5.4)	<.001	−0.1 (−1.7 to 1.5)	.93
CKD-EPI_WRC_ − iGFR	−1.7 (−2.5 to −0.9)	<.001	1.7 (−0.5 to 3.9)	.12	1.3 (−0.3 to 2.9)	.11	−1.4 (−2.8 to 0.1)	.07	−1.3 (−2.7 to 0.2)	.09	−1.9 (−3.4 to −0.5)	.009	−5.9 (−7.3 to −4.5)	<.001
CKD-EPI_CYS_ − iGFR	5.6 (4.6 to 6.6)	<.001	6.9 (4.0 to 9.7)	<.001	6.2 (4.1 to 8.3)	<.001	4.3 (2.4 to 6.2)	<.001	5.4 (3.5 to 7.4)	<.001	7.5 (5.7 to 9.4)	<.001	4.0 (2.2 to 5.9)	<.001

Abbreviations: CKD, chronic kidney disease; CKD-EPI_CYS_, cystatin C–based CKD-EPI eGFR; CKD-EPI_RC_, creatinine-based CKD-EPI eGFR calculated with the race coefficient; CKD-EPI_WRC_, creatinine-based CKD-EPI eGFR calculated without the race coefficient; eGFR, estimate glomerular filtration rate; GFR, glomerular filtration rate; iGFR, iothalamate GFR; NA not applicable.

^a^ Mean (SD) measurements were estimated as the intercept and residual standard deviation from an intercept-only linear mixed model with random intercepts to account for within-person correlation.

^b^ Mean differences (95% CIs) in measurements were estimated from an intercept-only linear mixed model with random intercepts to account for within-person correlation; corresponding *P* values test whether the mean difference in GFR was different from 0.

### Association of eGFR Estimating Equation With Time to an eGFR Less Than 20 mL/min/1.73 m^2^

Among the 1658 self-identified Black participants in this study, the median number of creatinine-based eGFR measurements was 8 (IQR, 4-12) and the median number of cystatin C-based eGFR measurements was 8 (IQR, 4-11). Among the 1616 participants with an eGFR (CKD-EPI_RC_) of at least 20 mL/min/1.73 m^2^ at study entry, 462 (28.6%) achieved an eGFR of less than 20 mL/min/1.73 m^2^ when using the race adjustment to calculate eGFR, and an additional 127 (7.9%), or 589 (36.4%) participants in total, would have achieved an eGFR less than 20 mL/min/1.73 m^2^ if the race adjustment were not used (Table 3 and eTable 1 in the Supplement). When CKD-EPI_CYS_ was used, 448 participants achieved the outcome. The median follow-up time was 4.4 years (IQR, 1.2-10.3 years). Not using the race coefficient in the creatinine-based CKD-EPI equation was associated with a 35% (95% CI, 29%-41%; *P* < .001) higher instantaneous risk of achieving an eGFR less than 20 mL/min/1.73 m^2^ compared with the CKD-EPI_RC_ equation. This association was significantly stronger among those with lower baseline eGFR. The estimated median time to achievement of an eGFR less than 20 mL/min/1.73 m^2^ from study enrollment was 13.9 years (95% CI, 13.0-13.9 years) when the race coefficient was used compared with 12.0 years (95% CI, 10.9-13.0 years) when it was not used (Figure). There was no significant difference in time to achievement of an eGFR less than 20 mL/min/1.73 m^2^ when CKD-EPI_CYS_ was used compared to CKD-EPI_RC_ (Table 3).

**Table 3.** Association of eGFR Calculation Method With Time to Achievement of an eGFR Less Than 20 mL/min/1.73 m^2^

	Overall	Baseline eGFR (CKD-EPI_RC_), mL/min/1.73 m^2^			
		<30	30 to <45	45 to <60	≥60
CKD-EPI_RC_					
No. at risk^a^	1616	278	605	498	235
No. of events	462	158	197	87	20
Incidence rate, per 100 person-years (95% CI)	4.8 (4.4 to 5.3)	22.2 (18.6 to 25.8)	6.2 (5.4 to 7.1)	2.4 (1.9 to 2.8)	1.0 (0.6 to 1.4)
CKD-EPI_WRC_					
No. at risk^a^	1616	278	605	498	235
No. events	589	199	253	112	25
Incidence rate, per 100 person-years (95% CI)	6.5 (6 to 7.1)	41.2 (33.1 to 49.2)	8.6 (7.5 to 9.6)	3.1 (2.6 to 3.7)	1.3 (0.8 to 1.8)
CKD-EPI_CYS_					
No. at risk^a^	1616	278	605	498	235
No. of events	448	155	189	87	17
Incidence rate, per 100 person-years (95% CI)	4.9 (4.4 to 5.3)	22.9 (18.5 to 27.2)	6.2 (5.3 to 7.0)	2.4 (2.0 to 2.9)	0.9 (0.5 to 1.3)
Difference in incidence rate, per 100 person-years (95% CI)^b^					
CKD-EPI_WRC_ – CKD-EPI_RC_	1.7 (1.4 to 2.0)	19.0 (13.3 to 24.7)	2.3 (1.8 to 2.9)	0.8 (0.5 to 1.0)	0.3 (0.0 to 0.5)
CKD-EPI_CYS_ – CKD-EPI_RC_	0.0 (−0.3 to 0.4)	0.7 (−2.6 to 3.9)	−0.1 (−0.7 to 0.6)	0.1 (−0.3 to 0.5)	−0.1 (−0.6 to 0.3)
Hazard ratio^c^					
CKD-EPI_WRC_ vs CKD-EPI_RC_	1.35 (1.29 to 1.41)^d^	1.81 (1.61 to 2.03)	1.38 (1.28 to 1.48)	1.32 (1.19 to 1.46)	1.26 (1.03 to 1.53)
CKD-EPI_CYS_ vs CKD-EPI_RC_	1.01 (0.94 to 1.08)^e^	1.02 (0.89 to 1.18)	1.00 (0.9 to 1.11)	1.05 (0.89 to 1.25)	0.89 (0.56 to 1.42)

Abbreviations: CKD, chronic kidney disease; CKD-EPI_CYS_, cystatin C–based CKD-EPI eGFR; CKD-EPI_RC_, creatinine-based CKD-EPI eGFR calculated with the race coefficient; CKD-EPI_WRC_, creatinine-based CKD-EPI eGFR calculated without the race coefficient; eGFR, estimated glomerular filtration rate.

^a^ Includes all participants with an eGFR (CKD-EPI_RC_) of at least 20 mL/min/1.73 m^2^ at study entry.

^b^ Difference in incidence rates per 100 person-years without minus with the race coefficient.

^c^ Instantaneous risk of an eGFR less than 20 mL/min/1.73 m^2^ associated with use of the race coefficient.

^d^ *P* < .001.

^e^ *P* = .85.

**Figure.**
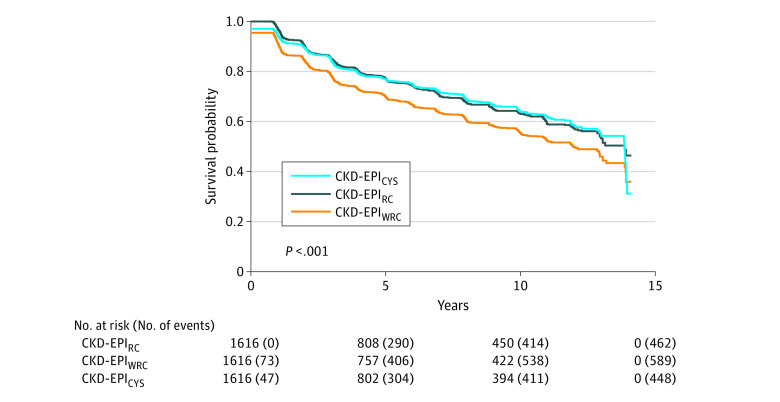
Kaplan-Meier Estimates of Time to Achievement of an Estimated Glomerular Filtration Rate (eGFR) Less Than 20 mL/min/1.73 m^2^ CKD-EPI_CYS_ indicates cystatin C–based CKD-EPI eGFR; CKD-EPI_RC_, creatinine-based CKD-EPI eGFR calculated with the race coefficient; CKD-EPI_WRC_, creatinine-based CKD-EPI eGFR calculated without the race coefficient.

### Association of the eGFR Estimating Equation With Time to an eGFR Less Than 30 mL/min/1.73 m^2^

There were 1338 participants with an eGFR (CKD-EPI_RC_) of at least 30 mL/min/1.73 m^2^ at study entry; of these, 746 achieved an eGFR of less than 30 mL/min/1.73 m^2^ if the race coefficient was not used compared with 579 achieving this outcome when the race coefficient was used to calculate eGFR and 617 when CKD-EPI_CYS_ was used (eTable 3 in the Supplement). The median follow-up time was 2.2 years (IQR, 0.0-7.9 years). Not using the race coefficient in the creatinine-based CKD-EPI equation was associated with a 52% (95% CI, 45%-59%) higher risk of achieving an eGFR less than 30 mL/min/1.73 m^2^ compared with CKD-EPI_RC_, with a difference in median time to event of 3.6 years (eFigure 3 in the Supplement); this association was strongest among participants with lower baseline eGFR. Use of CKD-EPI_CYS_ was associated with an 11% (95% CI, 5%-18%) higher risk of achieving an eGFR less than 30 mL/min/1.73 m^2^ compared with use of CKD-EPI_RC_.

## Discussion

In this cohort study of self-identified Black participants with CKD, use of the race coefficient in the creatinine-based CKD-EPI equation was associated with an eGFR that was a mean of 4.8 mL/min/1.73 m^2^ higher than when no race coefficient was used. Not using the race coefficient to estimate GFR (vs the standard race-based calculation) was also associated with a 35% higher risk of achieving an eGFR less than 20 mL/min/1.73 m^2^, with a decrease in median time to achievement of an eGFR less than 20 ml/min/1.73 m^2^ of 1.9 years. Thus, the biases in race-based GFR estimates, while numerically modest, may be associated with delays in potential preemptive transplant referral and eligibility among Black patients with CKD.

In our analysis, we found that there was poorer correspondence between iGFR and the eGFR using the CKD EPI equation with the race coefficient vs without the race coefficient. The eGFR with the race coefficient was higher than iGFR at lower ranges of iGFR (near the threshold for kidney transplant referral). In a previous study of the cohort assessed with the CKD-EPI equation,^22^ investigators found that across the range of eGFR, not including the race coefficient was associated with greater differences between the eGFR and iGFR compared with inclusion of the race coefficient. A possible explanation for the difference between the findings of that study and our study is that we presented the difference between eGFR and iGFR across the range of measured iGFR (and not eGFR as was done in the previous report). Further confirmatory studies, particularly using ancestry data, may provide more data on this issue.

Although the differences in eGFR with and without the race coefficient were numerically modest, in longitudinal analyses, the use of eGFR without the race coefficient was associated with a 35% higher risk of reaching the transplant eGFR threshold and a shorter median time to the end point of 1.9 years. Our results are similar to those of a recent analysis of more than 56 000 patients,^23^ in which investigators found that 3.1% of patients would be reassigned from an eGFR greater than 20 mL/min/1.73 m^2^ to an eGFR less than 20 mL/min/1.73 m^2^ with removal of the race coefficient.^23^ The small differences in eGFR and iGFR may be less meaningful in terms of health outcomes when inflexible clinical thresholds are not applied.^5^ However, in the scenario of potential access to possible preemptive kidney transplant, even a delay of 1 year for referral can have a significant effect on a patient’s life. With each year, there is rapid deterioration of health status, which may further limit eligibility because healthier patients are more likely to be placed on the waiting list.^24^

Currently, United Network for Organ Sharing criteria for kidney transplant listing do not specify a particular method for either estimating or measuring GFR. Alternative approaches to determine transplant eligibility have been proposed. One possibility is the use of cystatin C to estimate GFR rather than serum creatinine. However, in our analysis, we did not find significant differences in time to achievement of an eGFR less than 20 mL/min/1.73 m^2^ with use of cystatin C eGFR compared with creatinine-based eGFR with the race coefficient. Another potential approach is for nephrologists and transplant programs to use direct measurement of iGFR to guide such clinically important decisions such as kidney transplant eligibility. However, this may pose an additional barrier for kidney transplant referral owing to logistical issues involved with measuring iGFR routinely.

The dilemma on whether to include the race coefficient in GFR estimation has been highlighted in the national media and medical community.^5,25^ In response, a task force from the American Society of Nephrology and the National Kidney Foundation has been convened to provide recommendations on this important yet complicated issue. We recognize that removing the race coefficient may lead to other unintended effects; thus, the risks, benefits, and alternatives must be carefully considered.

### Strengths and Limitations

This study has strengths. Our study used a multicenter study population with iGFR and longitudinal measures of eGFR with both creatinine and cystatin C, the timing of which were unlikely to be confounded by clinical indication. This study also has limitations. We could not infer whether all patients in this population would have been eligible for a kidney transplant owing to the presence of other comorbidities. In addition, the present analysis is based on data from a research cohort of patients with CKD and thus may not reflect clinical practice in which patients may have more frequent monitoring of eGFR. In addition, the present analysis included subgroups of patients, which may further limit generalizability to the population with CKD.

## Conclusions

In this cohort study of self-reported Black participants with CKD, inclusion of the race coefficient in the estimation of GFR was associated with greater bias in GFR estimation and with delayed kidney transplant eligibility. These findings suggest that nephrologists and transplant programs should be cautious when using current estimating equations for GFR to determine kidney transplant eligibility.

## Notes

Supplement. **eFigure 1.** STROBE Flow Diagram of Participants **eFigure 2.** Correspondence of eGFR Bias With Gold-Standard iGFR Measurements **eFigure 3.** Kaplan-Meier Estimates of Time to eGFR <30 mL/min/1.73m^2^, With and Without Use of Race Coefficient **eTable 1.** Baseline Characteristics of Self-identified Black Participants in CRIC, by Analytic Subset **eTable 2.** Correspondence of eGFR and iGFR in CRIC, Among All Self-identified Black Participants With iGFR 15 - <45 mL/min/1.73m^2^, Overall and by BMI Category **eTable 3.** Association of eGFR Calculation Method and Time to eGFR <30 mL/min/1.73m^2^
