# Supplementary material for: Association of the Estimated Glomerular Filtration Rate With vs Without a Coefficient for Race With Time to Eligibility for Kidney Transplant
Source: JAMA Netw Open. 2021 Jan 14;4(1):e2034004. doi: 10.1001/jamanetworkopen.2020.34004 (PMC7809586; doi:10.1001/jamanetworkopen.2020.34004)
Supplement: Supplement. — eFigure 1. STROBE Flow Diagram of Participants eFigure 2. Correspondence of eGFR Bias With Gold-Standard iGFR Measurements eFigure 3. Kaplan-Meier Estimates of Time to eGFR <30 mL/min/1.73m2, With and Without Use of Race Coefficient eTable 1. Baseline Characteristics of Self-identified Black Participants in CRIC, by Analytic Subset eTable 2. Correspondence of eGFR and iGFR in CRIC, Among All Self-identified Black Participants With iGFR 15 - <45 mL/min/1.73m2, Overall and by BMI Category eTable 3. Association of eGFR Calculation Method and Time to eGFR <30 mL/min/1.73m2 [file jamanetwopen-e2034004-s001.pdf]

Supplemental Online Content

Zelnick LR, Leca N, Young B, Bansal N. Association of the estimated glomerular filtration rate with vs without a coefficient for race with time to eligibility for kidney transplant. *JAMA Netw Open.* 2021;4(1):e2034004. doi:10.1001/jamanetworkopen.2020.34004

eFigure 1. STROBE Flow Diagram of Participants

eFigure 2. Correspondence of eGFR Bias With Gold-Standard iGFR Measurements

eFigure 3. Kaplan Meier Estimates of Time to eGFR <30 mL/min/1.73m^2, With and Without Use of Race Coefficient

eTable 1. Baseline Characteristics of Self-identified Black Participants in CRIC, by Analytic Subset

eTable 2. Correspondence of eGFR and iGFR in CRIC, Among All Self-identified Black Participants With iGFR 15 - <45 mL/min/1.73m^2, Overall and by BMI Category

eTable 3. Association of eGFR Calculation Method and Time to eGFR <30 mL/min/1.73m^2

This supplemental material has been provided by the authors to give readers additional information about their work.

© 2021 Zelnick LR et al. *JAMA Network Open.*

eFigure 1. STROBE flow diagram of participants

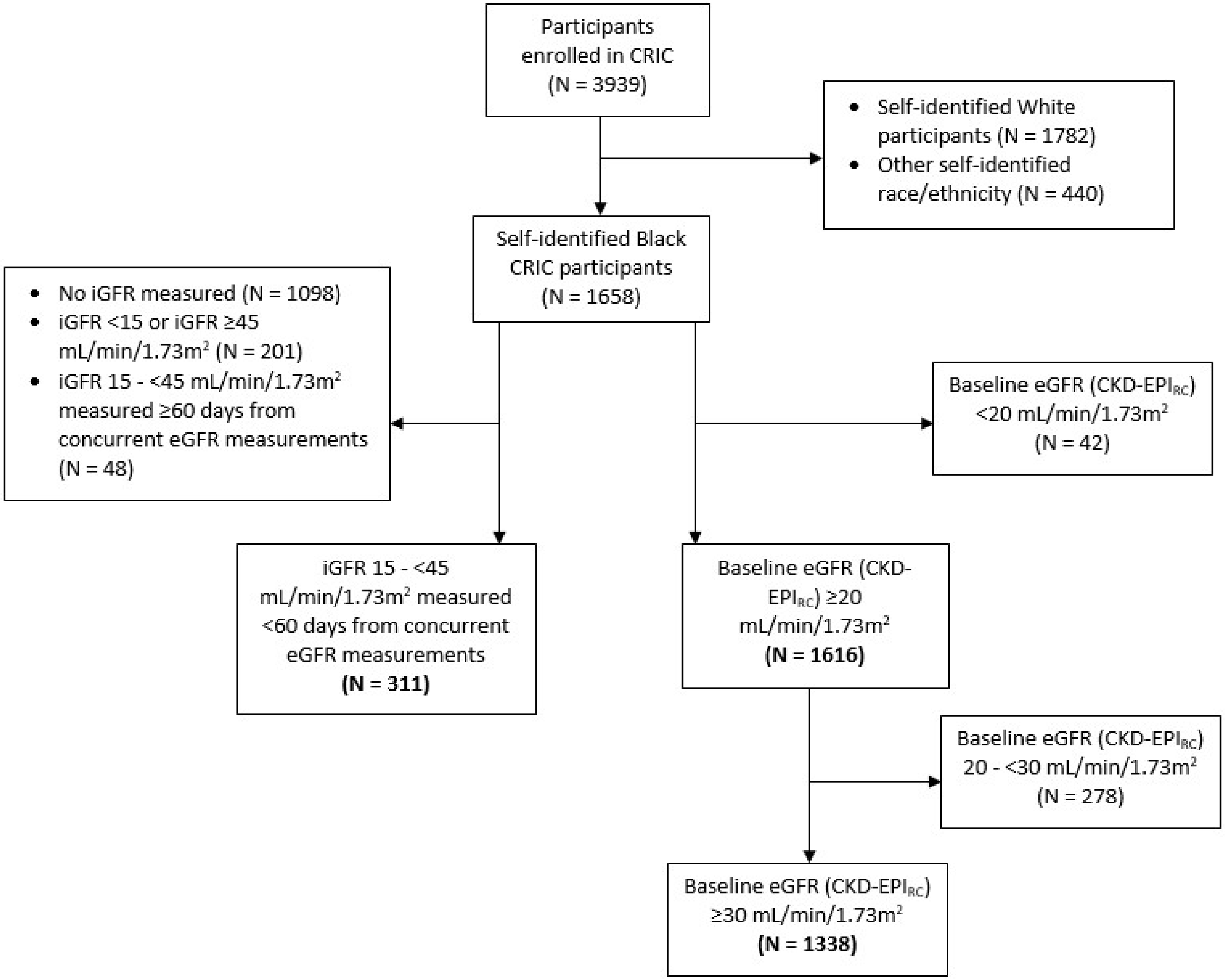

eGFR (CKD-EPI$_{RC}$) = creatinine-based Chronic Kidney Disease Epidemiology Collaboration estimated glomerular filtration rate calculated with race coefficient; iGFR = iothalamate glomerular filtration rate

© 2021 Zelnick LR et al. *JAMA Network Open.*

eFigure 2. Correspondence of eGFR bias with iGFR measurements

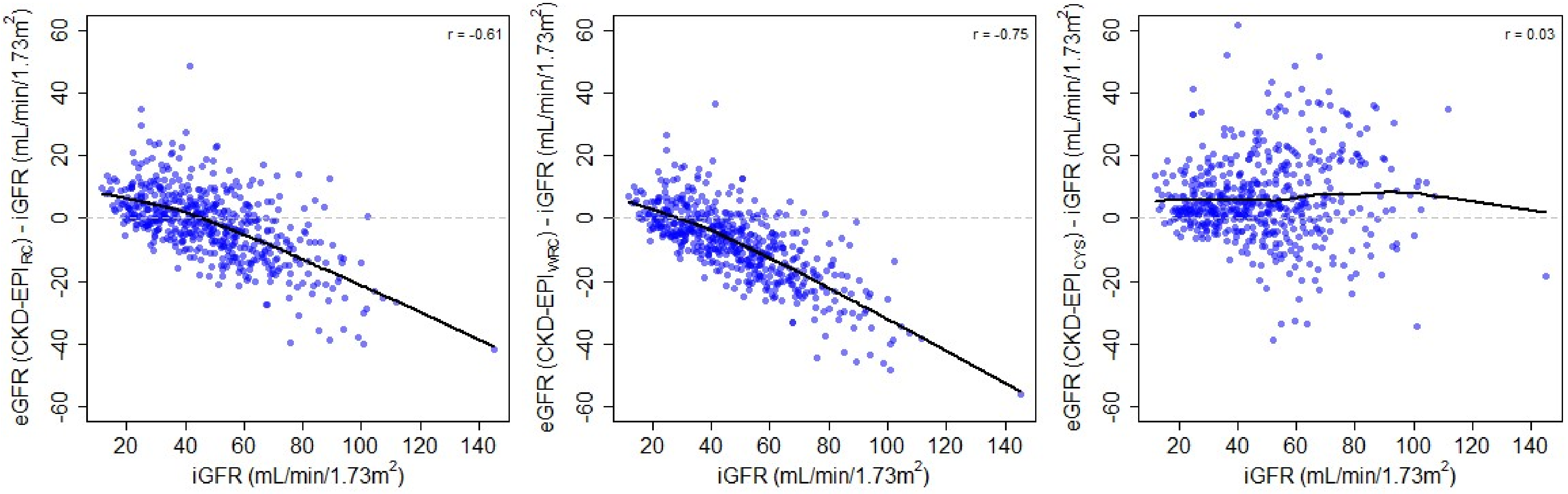

CKD-EPI$_{RC}$ = creatinine-based CKD-EPI eGFR calculated with race coefficient; CKD-EPI$_{WRC}$ = creatinine-based CKD-EPI eGFR calculated without the race coefficient; CKD-EPI$_{CYS}$ = cystatin-C-based CKD-EPI eGFR

© 2021 Zelnick LR et al. *JAMA Network Open.*

eFigure 3. Kaplan Meier estimates of time to eGFR <30 mL/min/1.73m²

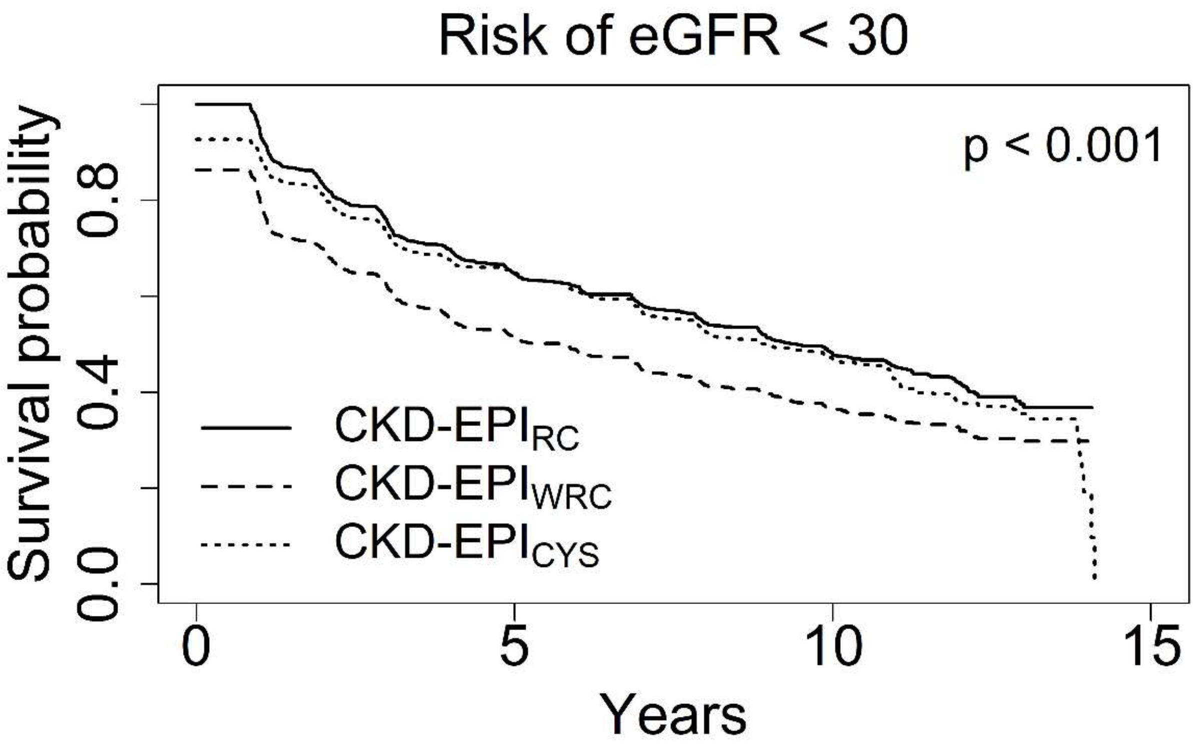

Number at risk (number of events)

CKD-EPI$_{RC}$	1338 (0)	618 (394)	340 (533)	0 (579)
CKD-EPI$_{WRC}$	1338 (183)	512 (584)	275 (713)	0 (746)
CKD-EPI$_{CYS}$	1338 (98)	641 (409)	301 (564)	0 (617)

CKD-EPI$_{RC}$ = creatinine-based CKD-EPI eGFR calculated with race coefficient; CKD-EPI$_{WRC}$ = creatinine-based CKD-EPI eGFR calculated without the race coefficient; CKD-EPI$_{CYS}$ = cystatin-C-based CKD-EPI eGFR

© 2021 Zelnick LR et al. *JAMA Network Open.*

eTable 1. Baseline characteristics of self-identified Black participants in CRIC, by analytic subset

	Participants experiencing CKD-EPI$_{RC}$ eGFR < 20 mL/min/1.73m^2 during follow-up* (N = 462)	Participants not experiencing CKD-EPI$_{RC}$ eGFR < 20 mL/min/1.73m^2 during follow-up* (N = 1154)	Participants with ≥ 1 iGFR measurement of 15 - <45 mL/min/1.73m^2 (N = 311)
Follow-up time (years)			
Mean (SD)	10.4	9.5 (4.3)	10.0 (3.8)
Median (IQR)	11.7 (8.1-13.2)	11.6 (5.9-12.9)	11.4 (7.3-13.1)
Age (years)	56.7 (10.8)	58.0 (10.5)	57.2 (12.0)
Male sex	224 (48)	565 (49)	162 (52)
Income			
≤ $20,000	182 (39)	450 (39)	137 (44)
$20,001 - $50,000	128 (28)	282 (24)	69 (22)
$50,001 - $10,000	53 (11)	161 (14)	40 (13)
> $100,000	14 (3)	47 (4)	15 (5)
Education			
Less than high school	132 (29)	295 (26)	85 (27)
High school graduate	89 (19)	269 (23)	66 (21)
Some college	163 (35)	393 (34)	100 (32)
College graduate or higher	78 (17)	197 (17)	60 (19)
Insurance status			
None	35 (8)	58 (5)	21 (7)
Medicaid/public aid	107 (23)	210 (18)	74 (24)
Any Medicare	154 (33)	330 (29)	97 (31)
VA/military/CHAMPUS	29 (6)	80 (7)	15 (5)
Private/commercial	57 (12)	130 (11)	28 (9)
History of CVD	188 (41)	428 (37)	111 (36)
History of hypertension	445 (96)	1053 (91)	301 (97)
History of MI	101 (22)	255 (22)	62 (20)
History of CHF	59 (13)	148 (13)	43 (14)
History of atrial fibrillation	93 (20)	228 (20)	50 (16)
History of stroke	73 (16)	151 (13)	42 (14)
Diabetes	271 (59)	556 (48)	169 (54)
Systolic blood pressure (mmHg)	136.9 (21.8)	131.0 (23.0)	134.7 (24.4)

© 2021 Zelnick LR et al. *JAMA Network Open.*

Diastolic blood pressure (mmHg)	75.2 (13.4)	73.1 (13.9)	73.9 (13.7)
BMI (kg/m^2)	34.0 (8.5)	33.3 (8.2)	33.3 (7.1)
Smoking	93 (20)	218 (19)	59 (19)
eGFR (CKD-EPI$_{RC}$) (mL/min/1.73m^2)	36.6 (11.9)	47.5 (14.3)	39.4 (12.3)
24-hour urine protein to creatinine ratio, median (IQR), (mg/g)	798 (237-2075)	105 (50-407)	415 (102-1717)
Serum albumin (g/dL)	3.8 (0.5)	4.0 (0.4)	3.8 (0.5)
Calcium (mg/dL)	9.1 (0.5)	9.2 (0.5)	9.2 (0.5)
Phosphate (mg/dL)	3.8 (0.6)	3.7 (0.6)	3.8 (0.7)

Entries are mean (SD) for continuous variables or N (%) for categorical variables, except as noted. CKD-EPI$_{RC}$ eGFR is the estimated glomerular filtration rate calculated using the creatinine-based Chronic Kidney Disease Epidemiology Collaboration equation including the race coefficient. Follow-up time is the time in years of follow-up in the CRIC study, with censoring for death, loss to follow-up, or end of study.
*Among participants with eGFR (CKD-EPI$_{RC}$) ≥ 20 mL/min/1.73m^2 at study entry.

© 2021 Zelnick LR et al. *JAMA Network Open.*

eTable 2. Correspondence of eGFR and iGFR in CRIC, among all self-identified Black participants with iGFR 15 - <45 mL/min/1.73m², overall and by BMI category

	Overall	BMI <30 kg/m²	BMI 30 - <35 kg/m²	BMI ≥35 kg/m²
N measurements	470	161	140	169
N unique individuals	311	105	93	113
**Mean (SD)**				
BMI	33.6 (1.8)	26.5 (0.8)	32.4 (1.2)	41.1 (2.1)
iGFR	31.8 (6.3)	32.1 (6.4)	31.4 (6.8)	31.8 (6.4)
$CKD\text{-}EPI_{RC}$	34.9 (7.8)	34.7 (5.8)	34.6 (10.5)	35.4 (7.5)
$CKD\text{-}EPI_{WRC}$	30.1 (6.7)	29.9 (5.0)	29.8 (9.0)	30.6 (6.5)
$CKD\text{-}EPI_{CYS}$	37.4 (8.3)	38.3 (7.4)	37.3 (9.9)	36.4 (8.9)
**Mean difference (95% CI)**				
$CKD\text{-}EPI_{RC}$ – $CKD\text{-}EPI_{WRC}$	4.8 (4.6, 4.9)	4.8 (4.5, 5.0)	4.7 (4.5, 5.0)	4.8 (4.6, 5.1)
p-value	< 0.001	< 0.001	< 0.001	< 0.001
$CKD\text{-}EPI_{RC}$ – $CKD\text{-}EPI_{CYS}$	-2.5 (-3.5, -1.5)	-3.4 (-4.9, -1.9)	-3.2 (-4.8, -1.7)	-1.1 (-2.6, 0.4)
p-value	< 0.001	< 0.001	< 0.001	0.16
$CKD\text{-}EPI_{RC}$ - iGFR	3.1 (2.2, 3.9)	2.8 (1.4, 4.3)	2.8 (1.3, 4.2)	3.5 (2.2, 4.9)
p-value	< 0.001	< 0.001	< 0.001	< 0.001
$CKD\text{-}EPI_{WRC}$ - iGFR	-1.7 (-2.5, -0.9)	-2.0 (-3.2, -0.7)	-2.0 (-3.3, -0.7)	-1.3 (-2.5, -0.1)
p-value	< 0.001	0.003	0.003	0.04
$CKD\text{-}EPI_{CYS}$ - iGFR	5.6 (4.6, 6.6)	6.4 (4.8, 8.0)	5.8 (4.1, 7.4)	4.6 (3.0, 6.2)
p-value	< 0.001	< 0.001	< 0.001	< 0.001

Mean (SD) estimated as the intercept and residual standard deviation from an intercept-only linear mixed model with random intercepts to account for correlation within-person. Mean differences and associated confidence intervals were similarly estimated from an intercept-only linear mixed model with random intercepts to account for correlation within-person; corresponding p-values test whether the mean difference in GFR is different from zero. $CKD\text{-}EPI_{RC}$ = creatinine-based CKD-EPI eGFR calculated with race coefficient; $CKD\text{-}EPI_{WRC}$ = creatinine-based CKD-EPI eGFR calculated without the race coefficient; $CKD\text{-}EPI_{CYS}$ = cystatin-C-based CKD-EPI eGFR; iGFR = iothalamate GFR

© 2021 Zelnick LR et al. *JAMA Network Open.*

eTable 3. Association of eGFR calculation method and time to eGFR <30 mL/min/1.73m^2

		By category of baseline eGFR (CKD-EPI$_{RC}$)		
	**Overall**	**30 - <45 mL/min/1.73m^2**	**45 - <60 mL/min/1.73m^2**	**≥ 60 mL/min/1.73m^2**
**CKD-EPI$_{RC}$**				
N at risk	1338	605	498	235
N events	579	361	182	36
IR, per 100 pys (95% bootstrap CI)	7.7 (7.0, 8.3)	15.9 (14.2, 17.7)	5.4 (4.7, 6.2)	1.9 (1.3, 2.4)
**CKD-EPI$_{WRC}$**				
N at risk	1338	605	498	235
N events	746	474	229	43
IR, per 100 pys (95% bootstrap CI)	11.8 (10.8, 12.8)	34.1 (29.6, 38.6)	7.6 (6.6, 8.5)	2.3 (1.7, 2.9)
**CKD-EPI$_{CYS}$**				
N at risk	1338	605	498	235
N events	617	380	196	41
IR, per 100 pys (95% bootstrap CI)	8.6 (7.9, 9.2)	17.5 (15.5, 19.5)	6.1 (5.3, 6.9)	2.2 (1.6, 2.9)
**Difference in IR, per 100 pys (95% bootstrap CI)**				
CKD-EPI$_{WRC}$ – CKD-EPI$_{RC}$	4.2 (3.6, 4.7)	18.2 (14.7, 21.6)	2.1 (1.6, 2.7)	0.4 (0.1, 0.7)
CKD-EPI$_{CYS}$ – CKD-EPI$_{RC}$	0.9 (0.4, 1.4)	1.6 (0.0, 3.1)	0.7 (0.1, 1.3)	0.4 (-0.1, 0.9)
**Hazard ratio**				
CKD-EPI$_{WRC}$ vs CKD-EPI$_{RC}$	1.52 (1.45, 1.59)	2.05 (1.88, 2.23)	1.38 (1.28, 1.49)	1.22 (1.07, 1.40)
p-value	< 0.001			
CKD-EPI$_{CYS}$ vs CKD-EPI$_{RC}$	1.11 (1.05, 1.18)	1.10 (1.01, 1.20)	1.13 (1.02, 1.25)	1.20 (0.94, 1.53)
p-value	< 0.001			

CKD-EPI$_{RC}$ = creatinine-based CKD-EPI eGFR calculated with race coefficient; CKD-EPI$_{WRC}$ = creatinine-based CKD-EPI eGFR calculated without the race coefficient; CKD-EPI$_{CYS}$ = cystatin-C-based CKD-EPI eGFR; IR = incidence rate; pys = person-years. Number at risk includes all participants with eGFR (CKD-EPI$_{RC}$) ≥30 mL/min/1.73m^2 at study entry. Difference in IR is the difference in incidence rates per 100 person-years without minus with the race coefficient. Hazard ratio is the instantaneous risk of eGFR <30 mL/min/1.73m^2 associated with use of the race coefficient.

© 2021 Zelnick LR et al. *JAMA Network Open.*
